# The potential benefit of metformin to reduce delirium risk and mortality: a retrospective cohort study

**DOI:** 10.18632/aging.204393

**Published:** 2022-11-17

**Authors:** Takehiko Yamanashi, Zoe-Ella EM Anderson, Manisha Modukuri, Gloria Chang, Tammy Tran, Pedro S. Marra, Nadia E. Wahba, Kaitlyn J. Crutchley, Eleanor J. Sullivan, Sydney S. Jellison, Katie R. Comp, Cade C. Akers, Alissa A. Meyer, Sangil Lee, Masaaki Iwata, Hyunkeun R. Cho, Eri Shinozaki, Gen Shinozaki

**Affiliations:** 1 Stanford University School of Medicine, Department of Psychiatry and Behavioral Sciences, Palo Alto, CA 94305, USA; 2 University of Iowa Carver College of Medicine, Department of Psychiatry, Iowa City, IA 52242, USA; 3 Tottori University Faculty of Medicine, Department of Neuropsychiatry, Yonago-Shi, Tottori, Japan; 4 University of Iowa Carver College of Medicine, Department of Emergency Medicine, Iowa City, IA 52242, USA; 5 University of Iowa College of Public Health, Department of Biostatistics, Iowa City, IA 52242, USA; 6 University of Iowa Carver College of Medicine, Department of Internal Medicine, Iowa City, IA 52242, USA

**Keywords:** delirium, metformin, diabetes mellitus, mortality, aging

## Abstract

Purpose: Metformin has been reported to improve age-related disorders, including dementia, and to lower mortality. This study was conducted to investigate whether metformin use lower delirium risk, as well as long-term mortality.

Methods: In this retrospective cohort study, previously recruited 1,404 subjects were analyzed. The relationship between metformin use and delirium, and the relationship between metformin use and 3-year mortality were investigated.

Main findings: 242 subjects were categorized into a type 2 diabetes mellitus (DM)-without-metformin group, and 264 subjects were categorized into a DM-with-metformin group. Prevalence of delirium was 36.0% in the DM-without-metformin group, and 29.2% in the DM-with-metformin group. A history of metformin use reduced the risk of delirium in patients with DM (OR, 0.50 [95% CI, 0.32 to 0.79]) after controlling for confounding factors. The 3-year mortality in the DM-without-metformin group (survival rate, 0.595 [95% CI, 0.512 to 0.669]) was higher than in the DM-with-metformin group (survival rate, 0.695 [95% CI, 0.604 to 0.770]) (p=0.035). A history of metformin use decreased the risk of 3-year mortality after adjustment for confounding factors (HR, 0.69 [95% CI, 0.48 to 0.98]).

Conclusions: Metformin use may lower the risk of delirium and mortality in DM patients.

## INTRODUCTION

Worldwide, we live in aging society, where growing numbers of older adults face significant risk for medical burdens including dementia, and more recently COVID-19 infection [1–3]. Delirium is also a severe medical illness common among older patients [4–7]. Delirium is associated with poor outcomes including extended length of stay, institutionalization after discharge from the hospital, and high mortality [4–6, 8]. The risk of delirium increases with age, and also with medical conditions such as infection including COVID-19 and after surgery [4–7, 9]. At present, there is no solid understanding of the pathogenesis of delirium, and thus we do not have therapeutic or preventative methods to effectively improve care of patients with delirium. It has been shown that commonly prescribed antipsychotic medications are not helpful for treatment or prevention [10–12], and novel viewpoints to investigate this devastating illness are warranted.

Additional major risk factor for delirium is baseline dementia [7]. Worse, after delirium, it is known that cognitive function further declines and dementia progression accelerates [13]. In the recent literature, there is evidence showing that type 2 diabetes mellitus (DM) may share a key process of pathophysiology with dementia. DM and high glucose levels have been tied to increased cognitive decline and risk for dementia [14–16]. This suggests that vascular and cellular damage induced by high blood glucose may mediate common pathological processes leading to dementia onset and progression [17]. These evidences suggest that DM also increases the risk of delirium potentially through common underline mechanisms that increase dementia risk. However, the literature is not consistent with regard to the association between DM and delirium despite the close relationship between delirium and dementia [18, 19].

Of interest, among various anti-diabetic medications, it has been shown that metformin may decrease the risk of various forms of dementia, including Alzheimer’s disease [20–23], although the results from various studies show inconsistency [24]. Because DM is associated with increased risk of dementia and cognitive decline, the association between anti-diabetic medication use and decreased risk of dementia was thought to be due to the better control of hyperglycemic mechanisms that could be a part of the pathogenesis of cognitive decline [25]. However, when metformin was compared to other anti-diabetic medications such as sulfonylureas (acetohexamide, chlorpropamide, glimepiride, glipizide, glyburide, tolazamide, and tolbutamide), the benefit for decreased risk of dementia and/or mortality was superior with metformin [22, 23, 26–28]. To date, although multiple studies have replicated data showing that metformin seems to have benefits for decreased risk of dementia and mortality, there is very limited data about the potential role of metformin and its association with delirium, mortality, and DM.

Thus, in this report we aimed to investigate the relationship between DM and delirium risk with a focus on the influence from metformin. We hypothesized that history of metformin use is associated with lower risk for delirium. We were also interested in testing if history of metformin use can alter one of the most important patient outcomes, mortality.

## MATERIALS AND METHODS

### Design

This report is based on our previous observational cohort study of delirium at the University of Iowa Hospitals and Clinics (UIHC) [29–33]. We conducted additional review of electronic medical records (EMRs) to gather information related to DM including body mass index (BMI), insulin use history, and metformin use history.

### Study participants

Our previously published work describes the details of study subjects and recruitment procedures [29–38]. Briefly, inclusion criteria were patients who were 18 years old or older from patients at UIHC either admitted as inpatients or visiting the emergency room. Exclusion criteria were patients whose goals of care were comfort measures only or ones with droplet/contact precautions. Patients meeting our study inclusion criteria were approached and enrolled if they or their legally authorized representative consented.

### DM and history of metformin and insulin use

Detailed metformin and insulin use history was obtained through an EMR review by the study team. Search terms such as “diabetes mellitus”, “metformin”, and “insulin” were used. Type 1 Diabetes mellitus as well as gestational diabetes were excluded from the DM group. Subjects with no history of metformin use at the time of study enrollment were classified into the metformin negative group. Other subjects, i.e., those who were taking at the time of study enrollment or had a history of metformin use before the enrollment, were classified in the metformin positive group. BMI at the time of enrollment was recorded.

### Clinical assessment and case definition

The procedures related to clinical data collection as well as definition of delirium status have been detailed in our reports published previously [29–38]. In brief, we reviewed hospital patient records and conducted patient interviews to collect medical history and demographic characteristics. Delirium scale instruments included the Confusion Assessment Method for Intensive Care Unit (CAM-ICU) [39], the Delirium Observation Screening Scale (DOSS) [40], and the Delirium Rating Scale—Revised-98 (DRS-R-98) [41]. The CAM-ICU and DRS were scored at the time of enrollment by trained research team members. As a part of the patient’s care, nursing staff recorded the DOSS score in the patient record. We defined patients’ delirium status based on CAM-ICU positive, DRS-R-98 ≥19, or DOSS ≥3, or clinical description in medical record showing the evidence of confusion or mental status change consistent with delirium [42]. When there were questionable cases with regard to delirium status, a board-certified consultation-liaison psychiatrist (G.S.) reviewed each case for final determination for classification.

### Assessment of mortality

All-cause mortality among the study participants were gathered from a review of medical records and obituary records as previously reported [30, 31, 33, 34, 38].

### Statistical analysis

The statistical software EZR was used for all statistical analyses reported here [43]. To compare the prevalence of delirium among the non-DM group, DM-with-metformin group, and DM-without-metformin group, the Chi-square test was used. To further test relationship between delirium and metformin use history in the DM group, logistic regression analysis was performed adjusting for covariates including age, sex, BMI, insulin use history, and dementia status. Furthermore, additional logistic regression analyses were performed separately for the subjects with dementia and subjects without dementia. In this logistic regression analyses, age, sex, Charlson Comorbidity Index (CCI), BMI, and insulin use history were included as covariates. For mortality analysis, Kaplan-Meier survival curves were used to visualize presentation of time to death, and log-rank statistics were used to assess significance of differences in 3-year mortality. First, we divided subjects into the following three groups: 1) non-DM group, 2) DM-without-metformin use, and 3) DM-with-metformin use. We also made subgroups divided by sex, age, presence of dementia, and presence of delirium to make Kaplan-Meier survival curves. To obtain hazard ratios (HRs) of death up to 3 years from study enrollment, we also used Cox proportional hazard regression models controlling for age, sex, CCI, BMI, insulin use history, and metformin use history using only DM subjects. Furthermore, additional Cox proportional hazard regression models controlling for same covariates were performed separately for the subjects with dementia and subjects without dementia. In addition, we performed propensity analyses. We divided subjects into two groups; non-dementia group and dementia group. The propensity for metformin use was determined using multivariable logistic regression analysis including five covariates; age, sex, CCI score, BMI, and insulin use history. The propensity scores were used to match metformin users to non-metformin users. 120 DM-with-metformin subjects were matched to 120 DM-without-metformin in non-dementia group and 28 DM-with-metformin subjects were matched to 28 DM-without-metformin in dementia group (Supplementary Figure 1). The p-values for comparisons among three groups were corrected by the Holm method. P-values <0.05 were considered statistically significant.

### Data availability

The data that support the findings of this study are available from the corresponding author, G.S., upon reasonable request.

## RESULTS

### Participant demographics

Data from a total of 1404 subjects recruited for our previous study [29–33] at UIHC between January 2016 and March 2020 were analyzed. The average patient age was 68.6 years (Standard Deviation, SD = 13.6), 48.7% were female, and 95.7% were non-Hispanic white per self-report; 898 patients were DM-negative, and 506 patients were DM-positive (Table 1). Among the 506 patients with DM, 264 had a history of metformin use. DM-without-metformin group had a significantly smaller BMI and less insulin use than DM-with-metformin group (mean [SD] BMI: DM-without-metformin group; 31.9 [9.4] vs DM-with-metformin group; 34.0 [9.2], t-test p = 0.01) (rate of insulin user: DM-without-metformin group; 45.0% vs DM-with-metformin group; 85.2%, chi-square test p < 0.001) (Table 1). Information about dementia, delirium status, Montreal Cognitive Assessment score, CCI, hospitalization unit, and length of hospital stay are shown in Table 1.

**Table 1:** Patient characteristics.

**Classification**	**All subjects**	**Diabetes**				**DM subjects**			
		**Non-DM**	**DM**			**Non-Met**	**Met**		
**N**	**1404**	**898**	**506**	**p**	**Statistical test**	**242**	**264**	**p**	**Statistical test**
Mean age — years old	68.6	68.0	69.7	0.03	t = -2.25	69.9	69.5	0.71	t = 0.37
SD	13.6	14.7	11.4			12.8	10.0		
Female sex (n)	684	222	462	0.008	χ2 = 7.13	110	112	0.55	χ2 = 0.36
%	48.7	43.9	51.4			45.5	42.4		
Race, White (n)	1344	862	482	0.61	χ2 = 0.27	229	253	0.67	χ2 = 0.18
%	95.7	96.0	95.3			94.6	95.8		
dementia (n)	221	129	92	0.07	χ2 = 3.27	45	47	0.91	χ2 = 0.01
%	15.7	14.4	18.2			18.6	17.8		
delirium (n)	413	249	164	0.07	χ2 = 3.20	87	77	0.13	χ2 = 2.35
%	29.4	27.7	32.4			36.0	29.2		
Mean MoCA	21.2	21.6	20.4	0.004	t = 2.88	20.7	20.1	0.40	t = 0.84
SD	6.7	6.7	6.7			6.5	6.9		
Mean CCI	3.4	2.6	5.0	<0.001	t = -2.71	5.0	4.9	0.95	t = 0.06
SD	3.0	2.6	3.0			3.2	2.8		
Mean BMI	30.4	28.9	32.9	<0.001	t = -8.78	31.9	34.0	0.01	t = -2.55
SD	8.5	7.6	9.3			9.4	9.2		
Insulin user		-	334			109	225	<0.001	χ2 = 89.1
%		-	66.0			45.0	85.2		
hospitalization unit				<0.001	χ2 = 37.8			0.08	χ2 = 8.19
General medicine (n)	854	516	338			151	187		
%	60.8	57.5	66.8			62.4	70.8		
ICU (n)	80	43	37			24	13		
%	5.7	4.8	7.3			9.9	4.9		
Emergency Department (n)	186	119	67			35	32		
%	13.2	13.3	13.2			14.5	12.1		
Orthopedics (n)	264	210	54			25	29		
%	18.8	23.4	10.7			10.3	11.0		
Others (n)	20	10	10			7	3		
%	1.4	1.1	2.0			2.9	1.1		
Mean LOS — days	9.1	8.5	10.1	0.003	t = -2.95	11.1	9.3	0.09	t = 1.69
SD	9.8	8.1	12.2			14.7	9.3		

Abbreviation: DM, type 2 diabetes mellitus; Met, Metformin; SD, Standard deviation; MoCA, Montreal Cognitive Assessment score; CCI, Charlson comorbidity index; BMI, Body mass index; LOS, length of hospital stay.

### Delirium, DM, and history of metformin use

**Figure 1:**
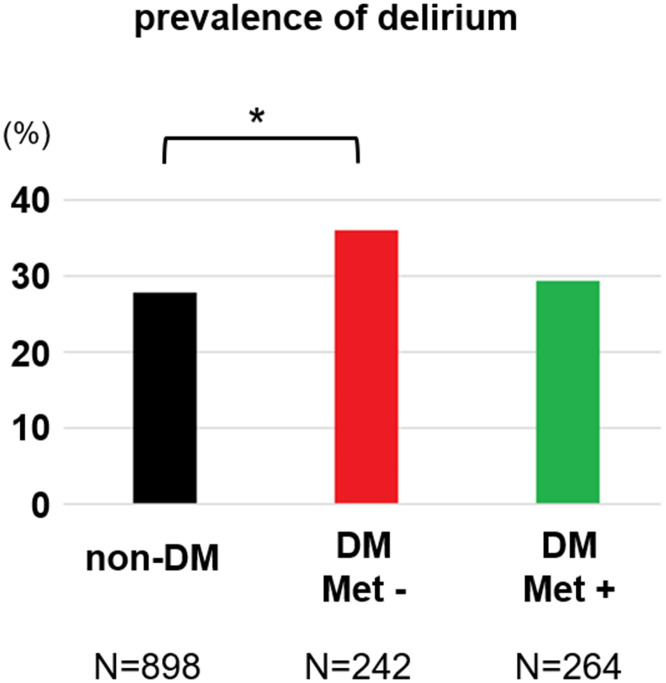
**Prevalence of delirium by comparing three patient groups based on their DM status and history of metformin use.** The Chi-square test corrected by Holm method showed significant difference between non-DM group and DM-without-metformin group (p=0.048).

The prevalence of delirium in the DM-without-metformin group (36.0%) was significantly higher than it was in the non-DM group (27.7%) (p = 0.048, Chi-square test corrected by Holm method) (Figure 1). The prevalence of delirium in the DM-with-metformin group (29.2%) was lower than it was in the DM-without-metformin group (36.0%), but this result was not statistically significant (p = 0.25, Chi-square test corrected by Holm method) (Figure 1). Logistic regression analysis using only DM subjects showed that a history of metformin use reduced the risk of delirium in patients with DM (OR = 0.50, 95% CI: 0.32–0.79, p = 0.003) even after controlling for age, sex, dementia status, BMI, and history of insulin use (Table 2). When logistic regression analyses were performed separately for the DM subjects with dementia and DM subjects without dementia, the results showed that metformin use history reduced risk of delirium both in non-dementia group (OR: 0.54, 95%CI: 0.32–0.91, p = 0.02) (Supplementary Table 1) and dementia group (OR: 0.40, 95%CI: 0.13–1.21, p = 0.11) (Supplementary Table 2) after controlling for age, sex, CCI, BMI, and history of insulin use, although dementia group did not reach statistically significant level likely due to reduced sample size.

**Table 2:** Result of the logistic regression in subjects with diabetes (N=506).

	**OR**	**95% CI**	**p-value**
Age	1.02	1.00 - 1.04	0.03
sex [male]	1.04	0.69 - 1.59	0.84
dementia	6.80	3.97 - 11.60	<0.001
BMI	0.97	0.95 - 1.00	0.04
Insulin User	2.85	1.71 - 4.74	<0.001
Metformin use history	0.50	0.32 - 0.79	0.003

Abbreviation: BMI, Body Mass Index.

### Mortality risk factors; benefit of history of metformin use

**Figure 2:**
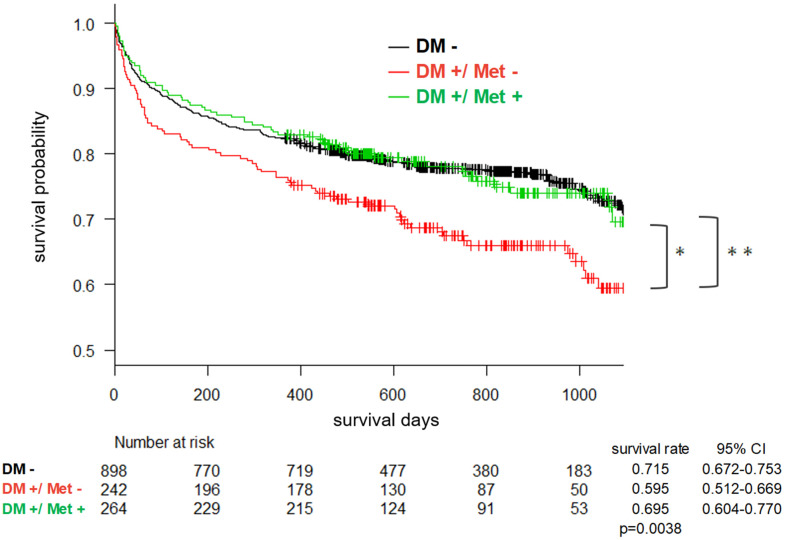
Kaplan-Meier cumulative survival curve over 3 years based on the three-group comparison.

First, 3-year mortality was compared between the following three groups: the non-DM group, DM-without-metformin group, and DM-with-metformin group. Mortality for the DM-without-metformin group (survival rate: 0.595, 95% CI: 0.512–0.669) was significantly higher than mortality for the non-DM group (survival rate: 0.715, 95% CI: 0.672–0.753) (p = 0.0036, log-rank test corrected by Holm method). On the other hand, mortality for the DM-with-metformin group (survival rate: 0.695, 95% CI: 0.604–0.770) was significantly lower than mortality for the DM-without-metformin group (p = 0.035, log-rank test corrected by Holm method). The mortality for DM-with-metformin group is almost exactly as good as that of non-DM patients (p = 0.91, log-rank test corrected by Holm method) (Figure 2). The same results were replicated in younger (age < 65) subgroup, aged (age ≥ 65) subgroup, female subgroup, male subgroup, non-dementia subgroup, and non-delirium subgroup (Supplementary Figures 2A–2D, 3A, 3C). Intriguing differences were found in dementia subgroup and delirium subgroup. Although it did not reach statistical significance, the mortality among DM patients who have used metformin were lower even when compared to that of non-DM group (Supplementary Figure 3B, 3D). Cox proportional hazard model showed that history of metformin use significantly decreased risk of 3-year mortality after adjustment for age, sex, CCI, BMI, history of insulin use, and delirium status (HR = 0.69, 95%CI: 0.48–0.98, p = 0.038) (Table 3). When cox proportional hazard models were performed separately for the DM subjects with dementia and DM subjects without dementia, the results showed that metformin use history did not reduce risk of mortality in non-dementia group (HR: 0.89, 95%CI: 0.59–1.35, p = 0.59) (Supplementary Table 3), but metformin exposure reduced risk of mortality among dementia group (HR: 0.40, 95%CI: 0.18–0.87, p = 0.02) (Supplementary Table 4). The GLOBAL tests for the proportional hazards assumptions were not statistically significant (Table 3 and Supplementary Tables 3, 4).

**Table 3:** Result of the Cox proportional hazard model in subjects with diabetes (N =506).

	**HR**	**95% CI**	**p-value**
Age	1.05	1.03 - 1.07	<0.001
sex male	1.37	0.97 - 1.92	0.07
CCI	1.15	1.10 - 1.20	<0.001
BMI	1.01	0.99 - 1.03	0.35
Insulin User	0.91	0.63 - 1.32	0.62
delirium	1.55	1.10 - 2.18	0.01
Metformin use history	0.69	0.48 - 0.98	0.04

Abbreviation: CCI, Charlson Comorbidity Index; BMI, Body Mass Index. Likelihood ratio test: p<0.001. Wald test: p<0.001. Score (logrank) test: p<0.001. GLOBAL test for the proportional hazards assumption: p=0.14.

### Results of propensity analyses

The characteristics of matched subjects are shown in Supplementary Table 5. The prevalence of delirium in the DM-with-metformin group was lower than one in the DM-without-metformin group both in non-dementia matched group (DM-without-metformin group: 37.5% vs DM-with-metformin group: 25.8%, Chi-square test p=0.07) and dementia matched group (DM-without-metformin group; 71.4% vs DM-with-metformin group; 60.7%, Chi-square test p=0.57), but without statistical significance (Supplementary Table 5). Logistic regression analysis showed a reduced risk of delirium by a history of metformin use both in non-dementia matched group (OR = 0.58, 95% CI: 0.34–1.01, p = 0.053) and dementia matched group (OR = 0.62, 95% CI: 0.20–1.89, p = 0.40), but without statistical significance. Mortality for the DM-with-metformin group was lower than mortality for the DM-without- metformin group both in non-dementia matched group (DM-without-metformin group; survival rate: 0.595, 95% CI: 0.476–0.700 vs DM-with-metformin group; survival rate: 0.658, 95% CI: 0.536–0.755, log-rank p = 0.29) and dementia matched group (DM-without- metformin group; survival rate: 0.382, 95% CI: 0.147– 0.617 vs DM-with-metformin group; survival rate: 0.709, 95% CI: 0.476–0.853, log-rank p = 0.07), but with statistical significance (Supplementary Figure 4). Cox proportional hazards model showed a reduced risk of mortality by a history of metformin use both in non-dementia matched group (HR = 0.78, 95% CI: 0.50–1.24, p = 0.29) and dementia matched group (OR = 0.44, 95% CI: 0.18–1.11, p = 0.08), but without statistical significance.

## DISCUSSION

This large-cohort study examined whether history of DM as well as metformin use are associated with delirium. Another investigation was if DM and history of metformin use are altering mortality risk. In the data presented here, higher prevalence of delirium and increased mortality were observed in DM patients without a history of metformin use compared to non-DM patients. On the other hand, DM patients with a history of metformin use showed lower prevalence of delirium in our data, and this could be a reason why past literature investigating relationship between DM and delirium was inconclusive, as most likely those subjects categorized with DM included those who were on metformin, and thus had less prevalence of delirium in average over DM patients with and without metformin use. Additionally, we found that a history of metformin use was associated with decreased risk of delirium and mortality even after adjusting for many potential confounding variables. In this study, we did perform several subgroup analyses and multiple regression analyses. They showed the same directional outcome showing that metformin was associated with reduced risk of delirium and long-term mortality, although several analyses did not reach statistically significant level likely because of the decreased sample size by sub-grouping. Our data indicate the potential positive benefit of metformin on delirium risk and mortality, providing insights about additional pathophysiological mechanisms of delirium and potential therapeutic and preventative opportunities for this devastating illness commonly seen among the aged population. However, it still remains unclear whether diabetes increases the risk of delirium or mortality as we have not analyzed the direct effect of diabetes on the risk of delirium and mortality with prospective clinical study including various important confounding factors.

The association observed here in this report between DM and delirium itself is not new, although previous data were mixed [18, 19]. However, the preventative role of metformin related to delirium is unique, and this is the first report showing such potential relationship. There has been little study investigating relationship between metformin and delirium. One study, which used the U.S. Food and Drug Administration adverse events reporting system (FAERS), reported metformin as a potential delirium-inducing drug, contrary to our result [44]. The discrepancy in these results might be due to methodological difference because the data in the FAERS are not designed to specifically investigate relationship between metformin and delirium but rather screening of large variety of commonly prescribed medications [44]. To solve this inconsistency, a future randomized clinical trial to test metformin for its effect on delirium risk would be of importance.

In addition, metformin has been reported to be beneficial for survival among various patient populations including cancer patients [45–47]. Our data presented here showed its potential benefit for survival regardless of delirium status. It is of importance to note that DM patients without a history of metformin use showing higher mortality was not simply due to their having a severe form of DM, because subjects in this group are defined as those who have never been on metformin, and they are not the group that was diagnosed with DM, treated with metformin first, and was switched to insulin due to their poor control of DM while they were on metformin. In fact, in our data set, BMI and insulin use ratio were lower in DM-without-metformin group than ones in DM-with-metformin group, suggesting that DM-without-metformin group had potentially less severe diabetes.

Basic research studies have shown that metformin appears to target a number of aging-related mechanisms [48, 49]. It was reported that metformin influenced pro-inflammatory cytokines such as IL-6 and TNF-alpha to suppress inflammation through modulating the NF-kB pathway [50, 51]. Furthermore, metformin was reported to activate AMPK and inhibits mTOR [52, 53], which is known to influence the aging process [49, 53, 54]. It is possible that these effects by metformin on inflammation and pathways involving AMPK and mTOR can decrease delirium risk and prolong patient’s life, although evidence for these effects in humans are limited.

Our data showed this potential benefit of metformin. The question is whether people without DM should start taking metformin. It is an important question that should be carefully explored, as metformin can have problematic side effects including vitamin B12 deficiency if taken for the long term [55]. Then the next question is if patients at risk for delirium such as those going through major surgery (e.g., cardiac, orthopedic, or neurosurgery) should take metformin even for a short period of time preoperatively and postoperatively. This needs to be answered by future clinical trials, and we believe these are worth conducting to improve our patient care.

### Limitations

We acknowledge the following limitations in this report. First, because history of metformin use was obtained from hospital records by retrospective chart review, it is possible to include false-positive cases when patients were prescribed with metformin but did not take it because of intolerable side effects or non-adherence to it, and false-negative situations such as metformin prescribed by other providers outside of our hospital network. Second, the definition of delirium is not based on the gold standard psychiatric assessment based on DSM-5 criteria [56]. However, our categorization methods have been effective enough to show discrete mortality risk based on our clinical classification of delirium as shown in our previous reports [29–33]. Also, Inouye et al. reported that retrospective chart review can capture delirium with reasonably good sensitivity and specificity [42]. Third, the definition of DM is based on chart review as well, although our classification of DM clearly differentiates mortality risk, supporting the validity of our approach. Fourth, we did not control for metformin dose or duration. Total dose information might be important because duration or dose of metformin can alter its effect, as a previous animal study showed [57]. Fifth, other anti-diabetic medication other than insulin were not recorded and incorporated in our investigation, as it has been repeatedly reported that when compared to metformin, other anti-diabetic medications did not show benefit for mortality [26–28]. Sixth, assessments for dementia and delirium were only performed at the time of recruitment, and they were not performed during the three-year follow-up period. The age of non-dementia group was about 7 years younger than dementia group. There is a possibility that part of non-dementia cohort could be classified in the dementia group couple years later. Part of this population might be detected as having dementia or delirium if assessments for dementia and delirium were performed during the follow-up period. It would be important to follow up over time to evaluate the long-term effects of metformin on delirium and dementia. It should be an important agenda for future study. Lastly, our data does not necessarily show causal relationship of metformin use and risk for delirium or mortality. However, metformin was used prior to the occurrence of delirium as well as death, suggesting a strong possibility of a beneficial effect from metformin in decreasing risk for delirium and increasing chance of survival. To address this question more precisely, prospective clinical trials are needed. Despite all these potential limitations, we found significant associations among metformin, DM, and delirium, as well as all-cause mortality.

## CONCLUSIONS

In this report, we showed the potential benefit of metformin in decreasing the risk of delirium and mortality in DM subjects.

## Supplementary Material

Supplementary Figures

Supplementary Tables
