# Supplementary material for: The potential benefit of metformin to reduce delirium risk and mortality: a retrospective cohort study
Source: Aging (Albany NY). 2022 Nov 17;14(22):8927–43. doi: 10.18632/aging.204393 (PMC9740381; doi:10.18632/aging.204393)
Supplement: Supplementary Figures [file aging-14-204393-s001.pdf]

SUPPLEMENTARY FIGURES

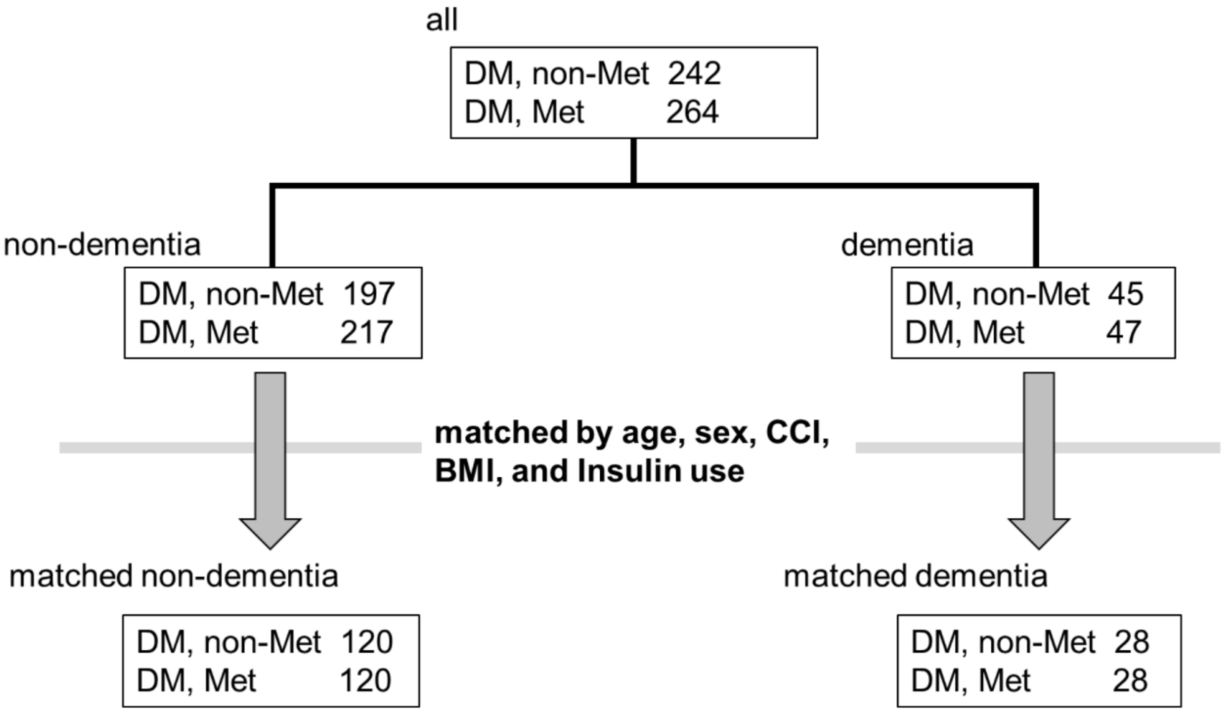

Supplementary Figure 1. Propensity score matching process.

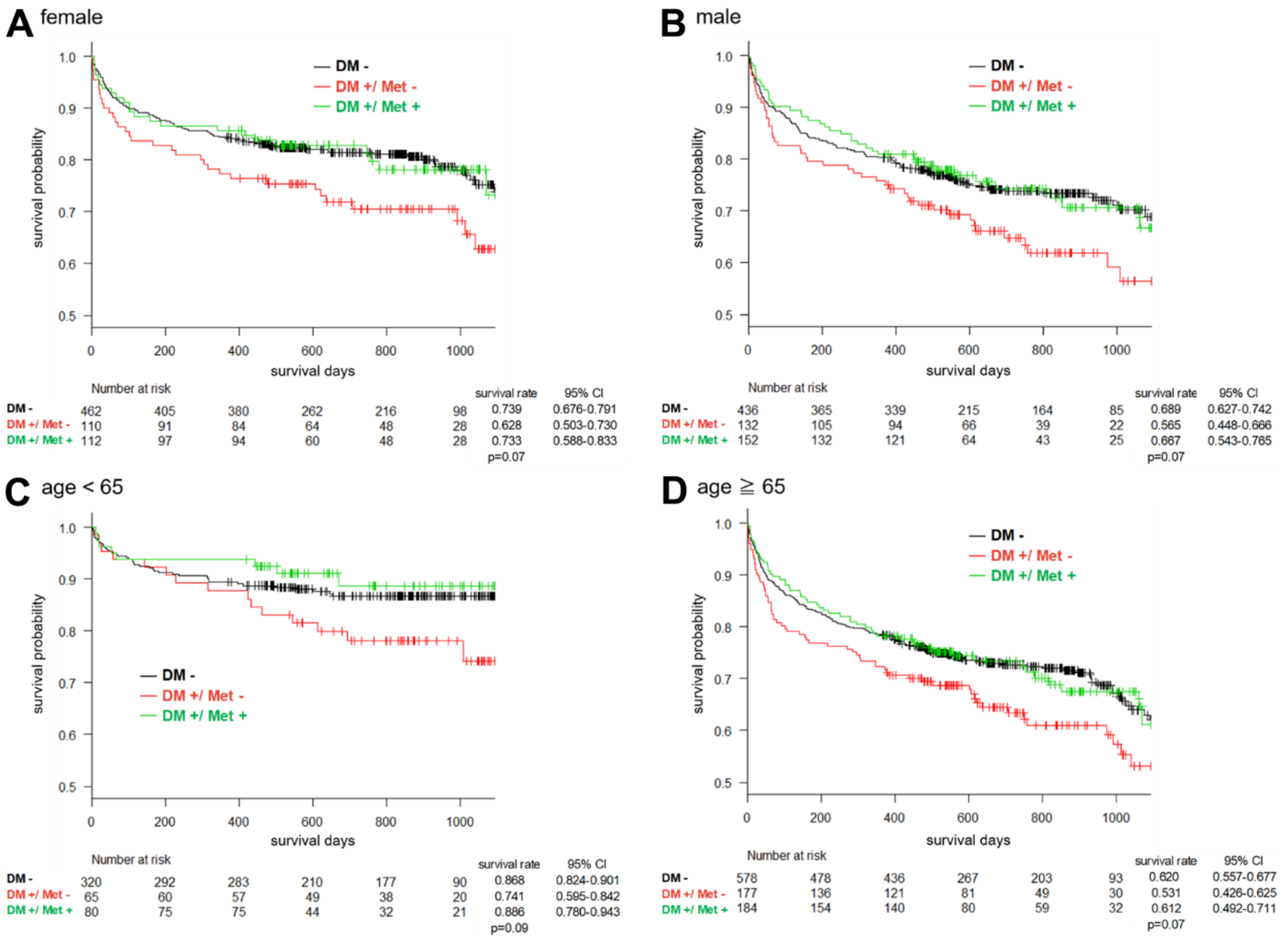

Supplementary Figure 2. Kaplan-Meier cumulative survival curve over 3 years based on (**A**) female only cohort, (**B**) male only cohort, (**C**) age <65 years cohort, (**D**) age ≥65 years cohort.

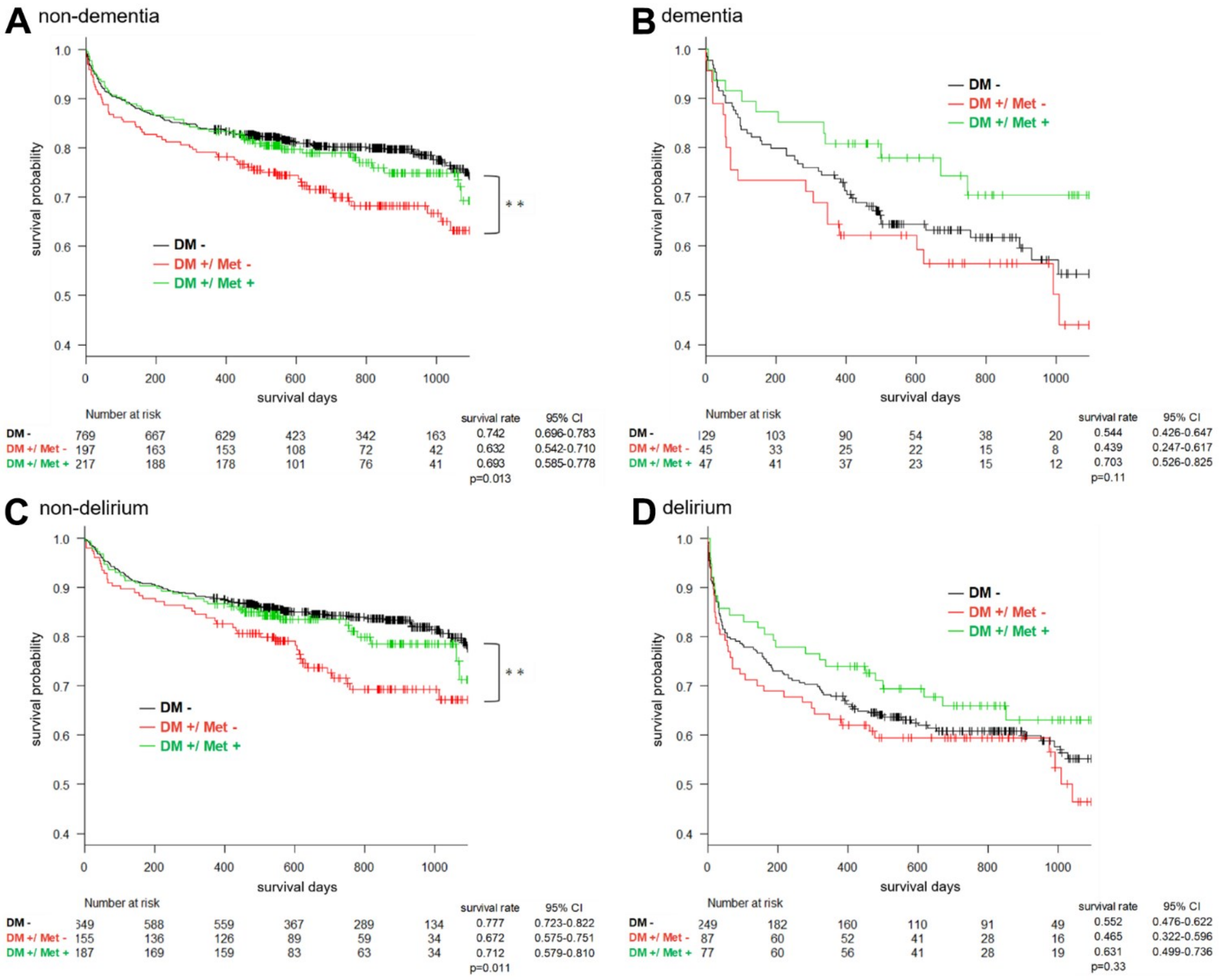

Supplementary Figure 3. Kaplan-Meier cumulative survival curve over 3 years based on (**A**) non-dementia cohort, (**B**) dementia cohort, (**C**) non-delirium cohort, (**D**) delirium cohort. ** p<0.01.

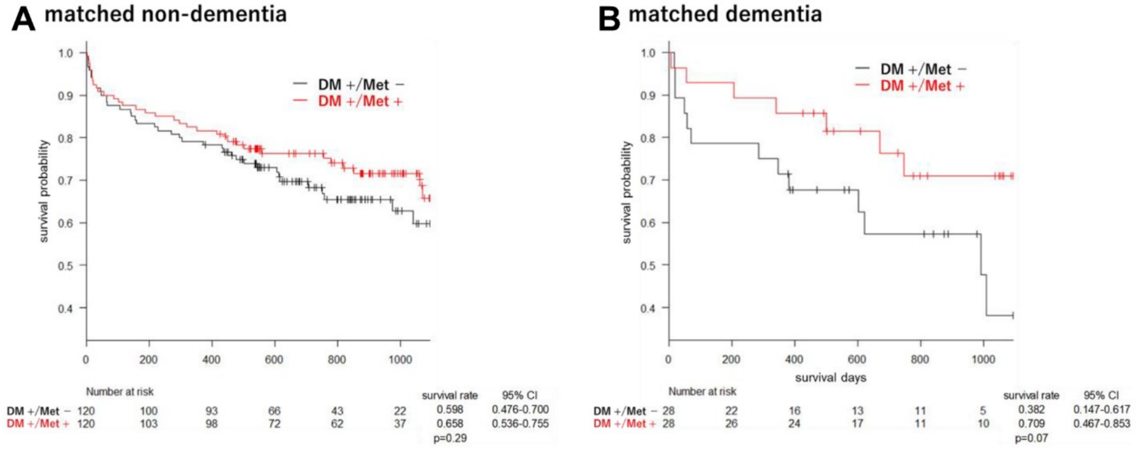

Supplementary Figure 4. Kaplan-Meier cumulative survival curve over 3 years based on (**A**) matched non-dementia cohort and (**B**) matched dementia cohort.
