# Supplementary material for: The potential benefit of metformin to reduce delirium risk and mortality: a retrospective cohort study
Source: Aging (Albany NY). 2022 Nov 17;14(22):8927–43. doi: 10.18632/aging.204393 (PMC9740381; doi:10.18632/aging.204393)
Supplement: Supplementary Tables [file aging-14-204393-s002.pdf]

SUPPLEMENTARY TABLES

Supplementary Table 1. Result of the logistic regression in non-dementia subjects with diabetes (N=414).

	OR	95% CI	p-value
Age	1.02	0.997 - 1.04	0.09
sex [male]	1.03	0.64 - 1.66	0.90
CCI	1.09	1.01 - 1.17	0.03
BMI	0.98	0.95 - 1.00	0.09
Insulin User	2.40	1.34 - 4.29	0.003
Metformin use history	0.54	0.32 - 0.91	0.02

Abbreviation: CCI, Charlson Comorbidity Index; BMI, Body Mass Index.

Supplementary Table 2. Result of the logistic regression in dementia subjects with diabetes (N=92).

	OR	95% CI	p-value
Age	1.01	0.95 - 1.06	0.84
sex [male]	0.98	0.35 - 2.71	0.97
CCI	1.11	0.94 - 1.32	0.22
BMI	0.93	0.86 - 0.998	0.04
Insulin User	4.30	1.35 - 13.7	0.01
Metformin use history	0.40	0.13 - 1.21	0.11

Abbreviation: CCI, Charlson Comorbidity Index; BMI, Body Mass Index.

Supplementary Table 3: Result of the Cox proportional hazard model in non-dementia subjects with diabetes (N=414).

	HR	95% CI	p-value
Age	1.05	1.03 - 1.07	<0.001
sex male	1.43	0.96 - 2.12	0.07
CCI	1.18	1.12 - 1.24	<0.001
BMI	1.01	0.98 - 1.03	0.62
Insulin User	0.77	0.50 - 1.19	0.23
delirium	2.10	1.42 - 3.13	<0.001
Metformin use history	0.89	0.59 - 1.35	0.59

Abbreviation: CCI, Charlson Comorbidity Index; BMI, Body Mass Index. Likelihood ratio test: p<0.001. Wald test: p<0.001. Score (logrank) test: p<0.001. GLOBAL test for the proportional hazards assumption: p=0.43.

Supplementary Table 4. Result of the Cox proportional hazard model in dementia subjects with diabetes (N=92).

	HR	95% CI	p-value
Age	1.07	1.02 - 1.12	0.005
sex male	1.00	0.50 - 2.02	>0.99
CCI	1.11	1.00 - 1.23	0.06
BMI	1.04	0.98 - 1.10	0.16
Insulin User	1.21	0.55 - 2.67	0.64
delirium	0.98	0.45 - 2.15	0.97
Metformin use history	0.40	0.18 - 0.87	0.02

Abbreviation: CCI, Charlson Comorbidity Index; BMI, Body Mass Index. Likelihood ratio test: p=0.03. Wald test: p=0.04. Score (logrank) test: p=0.03. GLOBAL test for the proportional hazards assumption: p=0.14.

Supplementary Table 5. Patient characteristics of matched subjects.

Classification	Non-dementia DM subjects				Dementia DM subjects			
	Non-Met	Met			Non-Met	Met		
N	120	120	p	Statistical test	28	28	p	Statistical test
Mean age — years	68.0	68.5	0.74	t = -0.33	75.1	75.9	0.75	t = -0.32
SD	12.4	10.9			9.3	9.1		
Female sex (n)	56	64	0.37	χ2 = 0.82	9	11	0.78	χ2 = 0.08
%	46.7	53.3			32.1	39.3		
Race, White (n)	112	116	0.37	χ2 = 0.79	27	27	>0.99	χ2 = 0.00
%	93.3	96.7			96.4	96.4		
delirium (n)	45	31	0.07	χ2 = 3.25	20	17	0.57	χ2 = 0.32
%	37.5	25.8			71.4	60.7		
Mean MoCA	21.8	20.8	0.24	t = 1.17	13.1	13.5	0.86	t = -0.17
SD	5.6	6.3			8.4	7.6		
Mean CCI	4.9	4.7	0.70	t = 0.38	5.9	5.5	0.52	t = 0.66
SD	3.0	2.8			3.0	2.3		
Mean BMI	32.1	32.6	0.69	t = -0.41	31.7	30.4	0.45	t = 0.77
SD	9.3	8.3			6.7	6.4		
Insulin user	90	91	>0.99	χ2 = 0.00	19	19	>0.99	χ2 = 0.00
%	75.0	75.8			67.9	67.9		
hospitalization unit			0.53	χ2 = 3.19			0.47	χ2 = 3.56
General medicine (n)	71	77			22	23		
%	59.2	64.2			78.6	82.1		
ICU (n)	13	6			2	0		
%	10.8	5.0			7.1	0.0		
Emergency Department (n)	22	22			1	2		
%	18.3	18.3			3.6	7.1		
Orthopaedics (n)	11	13			2	3		
%	9.2	10.8			7.1	10.7		
Others (n)	3	2			1	0		
%	2.5	1.7			3.6	0.0		
Mean LOS — days	11.8	9.3	0.13	t = 1.54	14.9	10.3	0.39	t = 0.87
SD	15.5	8.9			23.7	13.7		

Abbreviation: DM, type 2 diabetes mellitus; Met, Metformin; SD, Standard deviation; MoCA, Montreal Cognitive Assessment score; CCI, Charlson comorbidity index; BMI, Body mass index; LOS, length of hospital stay.
